# Computed Tomography Versus Pathologic Tumor Size in Resected Lung Tumors: High Correlation, Limited Agreement, and the Impact of Ground-Glass Opacity

**DOI:** 10.3390/tomography12050067

**Published:** 2026-05-11

**Authors:** Omer Yavuz, Reyhan Ertan, Muhammet Kertmen, Mehlika Iscan

**Affiliations:** Thoracic Surgery Department, Basaksehir Çam ve Sakura City Hospital, 34480 Istanbul, Turkey

**Keywords:** computed tomography, pathology, tumor size, agreement analysis, Bland–Altman analysis, ground-glass opacity, lung cancer, radiologic/pathologic correlation, T-category concordance

## Abstract

**Background:** Computed tomography (CT) is routinely used to estimate tumor size before lung resection, whereas pathologic examination provides the reference tissue-based measurement after surgery. This study aimed to compare CT-derived and pathologic tumor size and to evaluate correlation, agreement, proportional bias, clinically defined accuracy, and size-based T-category concordance, with particular attention to the effect of ground-glass opacity (GGO). **Methods:** This retrospective single-center study included 96 patients who underwent lung resection between January 2023 and December 2025 and had complete preoperative CT and pathologic tumor measurements. Maximum tumor diameter was defined as the largest of three orthogonal measurements for each modality. Correlation was assessed using Spearman’s rank correlation coefficient, reliability using the intraclass correlation coefficient (ICC), and agreement using Bland–Altman analysis. Proportional bias was evaluated by regression of the paired difference on the paired mean. Subgroup, size category, regression and size-based T-category concordance analyses were also performed. **Results:** CT and pathologic maximum diameters showed strong correlation (Spearman’s ρ = 0.952, *p* < 0.0001) and excellent reliability (ICC = 0.959, 95% CI, 0.939–0.973). The paired comparison was not statistically significant (*p* = 0.175), and the mean bias was −0.76 mm. However, the 95% limits of agreement ranged from −13.66 mm to +12.13 mm. Significant proportional bias was observed, with increasing CT underestimation as tumor size increased (slope = −0.093, *p* = 0.0014). In tumors with GGO, CT pathology differences shifted toward overestimation (+8.91 ± 7.30 mm vs. −1.64 ± 5.80 mm without GGO; *p* = 0.0003). Accuracy within ±5 mm and ±10 mm was 68.8% and 88.5%, respectively, but was lower in the GGO subgroup. CT-derived and pathology-derived size-based T-categories were concordant in 60 patients (62.5%), while pathology-based upstaging occurred in 23 patients (24.0%) and pathology-based downstaging in 13 patients (13.5%). **Conclusions:** CT-based tumor size showed strong overall correlation with pathologic measurements, but agreement at the individual patient level was more limited than correlation metrics alone would suggest. GGO and tumor size appeared to be important modifiers of measurement performance; however, the GGO-related findings should be interpreted cautiously because of the small subgroup size. These findings support cautious interpretation of CT-derived whole-lesion diameter, particularly in subsolid tumors and larger lesions.

## Simple Summary

Tumor size is an important parameter in lung cancer evaluation, and computed tomography (CT) is commonly used to estimate it before surgery. However, the size measured on CT may not always match the size measured on the resected pathologic specimen. In this study of 96 patients, CT-based and pathologic tumor size showed very strong overall correlation, but individual-level agreement was more limited. Differences were particularly notable in tumors with ground-glass opacity and in larger tumors, and these discrepancies also led to shifts in size-based T-category assignment in a substantial subset of patients. These results indicate that CT-derived whole-lesion diameter remains useful, but it should be interpreted with caution when used as a surrogate for pathologic tumor size.

## 1. Introduction

Tumor size is a central parameter in thoracic oncology because it affects radiologic assessment, surgical planning, and pathologic classification [1,2]. In routine practice, preoperative computed tomography (CT) is commonly used as the principal imaging-based estimate of tumor size, whereas pathologic examination of the resected specimen provides the reference tissue-based measurement [3,4].

However, correlation between two measurement methods does not necessarily indicate agreement [5]. Two methods may show a strong association across a cohort while still differing substantially at the individual patient level [6]. This distinction is particularly relevant when differences of only a few millimeters may influence clinical interpretation [7].

The aim of this study was to compare CT-derived tumor size with pathologic tumor size in a retrospective single-center cohort of patients who underwent lung resection. In addition to correlation and reliability, we evaluated agreement, systematic bias, proportional bias, and clinically defined measurement accuracy, with particular attention to the effect of ground-glass opacity (GGO).

## 2. Materials and Methods

### 2.1. Study Design and Patient Cohort

This retrospective single-center study was based on data from patients who underwent lung resection between January 2023 and December 2025. Patients with complete preoperative CT measurements and corresponding pathologic specimen measurements were included, yielding a final cohort of 96 patients.

### 2.2. Measurement Definitions

Tumor measurements were obtained retrospectively from the available preoperative CT images and the corresponding final pathology reports. CT-based tumor measurements were performed by the authors using a predefined approach based on three orthogonal tumor dimensions. For each case, the anteroposterior, lateral, and craniocaudal dimensions were recorded, and the maximum tumor diameter was defined as the largest of these three measurements. The recorded CT measurements were reviewed for consistency before analysis.

Pathologic tumor measurements were retrospectively extracted from the final routine pathology reports issued after surgical resection. For each case, three orthogonal pathology specimen dimensions were available, and pathologic maximum diameter was defined as the largest of these three measurements. No additional post hoc pathologic re-measurement was performed for this study.

Because dedicated segmentation-based CT volumetry was not consistently available in this retrospective cohort, volumetric assessment was not used as a primary study endpoint. Instead, as a secondary exploratory analysis, estimated ellipsoid tumor volume was calculated for both CT and pathology using the formula

**Figure 1:**
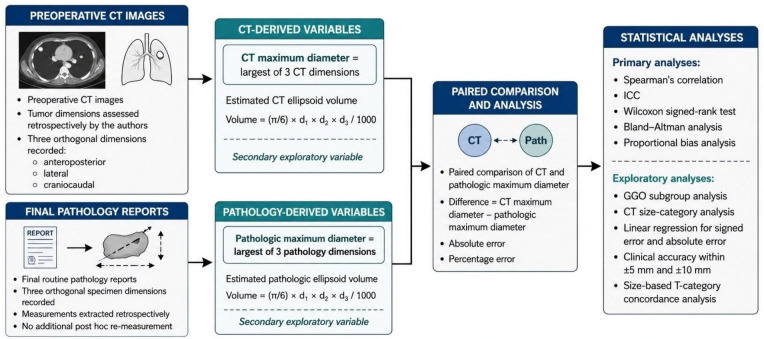
Schematic illustration of the measurement workflow used in this study. Preoperative CT images and final pathology reports provided three orthogonal tumor dimensions for each case. Maximum tumor diameter was defined as the largest recorded dimension for each modality, and estimated ellipsoid volume was used as a secondary exploratory variable.

Volume = (π/6) × d1 × d2 × d3/1000

with dimensions expressed in millimeters and volume expressed in cm^3^. These estimated volumes were used only for secondary correlation and reliability analyses. The measurement workflow is illustrated in Figure 1.

### 2.3. Study Variables

The primary agreement variable was the paired difference between CT and pathologic maximum diameter, defined as

Difference = CT maximum diameter − pathologic maximum diameter

Absolute error was defined as the absolute value of this difference. Percentage error was calculated relative to pathologic maximum diameter. GGO status was recorded as absent or present. Volume-derived variables were not used for subgroup analyses, regression models, or clinical accuracy thresholds.

### 2.4. Statistical Analysis

Continuous variables were summarized using the mean ± standard deviation and median with interquartile range, as appropriate. Distributional normality was assessed with the Shapiro–Wilk test. Because the main variables deviated from normality, nonparametric methods were used for the primary analyses.

Correlation between CT and pathologic measurements was assessed using Spearman’s rank correlation coefficient. Reliability and absolute agreement were further evaluated using the intraclass correlation coefficient (ICC), based on a two-way mixed-effects, absolute-agreement, single-measure model [8,9].

For the primary analysis, paired CT and pathologic maximum diameters were compared using the Wilcoxon signed-rank test. Agreement for maximum diameter was assessed with Bland–Altman analysis, including calculation of mean bias and 95% limits of agreement (LoA). Proportional bias for maximum diameter was evaluated by regression of the paired difference on the paired mean. Estimated ellipsoid volumes were analyzed as a secondary exploratory endpoint using Spearman’s correlation and ICC.

A size-based T-category concordance analysis was additionally performed using the maximum tumor diameter thresholds of the eighth edition of the TNM classification. CT maximum diameter was used to assign the CT-derived size-based T-category, and pathologic maximum diameter was used to assign the pathology-derived size-based T-category. The analysis was restricted to size descriptors and did not incorporate non-size T criteria. Concordance, pathology-based upstaging, and pathology-based downstaging rates were calculated and reported as counts and percentages.

Exploratory subgroup analyses were performed according to GGO status and CT-based tumor size category (≤20 mm, 20–50 mm, and >50 mm). Between-group comparisons were performed using the Mann–Whitney U test or Kruskal–Wallis test, as appropriate. Linear regression models were constructed using CT maximum diameter and GGO status as predictors of signed error and absolute error. Clinical accuracy was additionally assessed as the proportion of cases with CT pathology differences within ±5 mm and ±10 mm. A two-sided *p* value < 0.05 was considered statistically significant. Statistical analyses were performed using Python (version 3.10; Python Software Foundation, Beaverton, OR, USA).

Google Gemini 1.5 Pro (via Google Imagen 3) was used only for non-data-derived figures in the manuscript, including the graphical abstract and workflow diagram. No AI tools were used for the generation or modification of data-derived figures, primary data analysis, statistical calculations, or interpretation of the results. The authors reviewed, verified, and approved all AI-assisted outputs and take full responsibility for the content of this publication.

## 3. Results

### 3.1. Cohort Characteristics

A total of 96 patients were included. The mean CT maximum diameter was 39.24 ± 21.94 mm, and the mean pathologic maximum diameter was 40.00 ± 24.03 mm. Median values were 34.90 mm (IQR, 20.85–50.73) for CT and 35.50 mm (IQR, 20.00–55.75) for pathology. GGO was present in 8 patients (8.3%) and absent in 88 patients (91.7%). Cohort characteristics and overall measurement summary are presented in Table 1.

### 3.2. Correlation and Reliability

**Figure 2:**
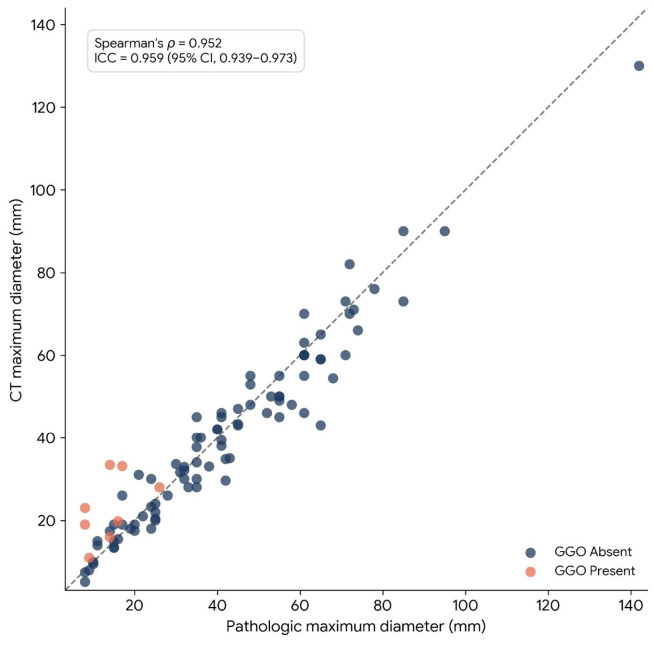
Scatter plot showing the relationship between CT maximum diameter and pathologic maximum diameter. Each point represents one patient and is colored according to ground-glass opacity (GGO) status. The dashed line indicates the line of identity (y = x). CT and pathologic maximum diameters showed strong correlation and excellent agreement at the cohort level (Spearman’s ρ = 0.952; ICC = 0.959, 95% CI, 0.939–0.973).

CT and pathologic maximum diameters showed a strong correlation (Spearman’s ρ = 0.952, *p* < 0.0001). The ICC for maximum diameter was 0.959 (95% CI, 0.939–0.973). The relationship between CT and pathologic maximum diameters is shown in Figure 2.

### 3.3. Secondary Volumetric Analysis

In a secondary exploratory volumetric analysis based on estimated ellipsoid volumes, CT and pathologic volumes showed a Spearman’s ρ of 0.968 (*p* < 0.0001), and the ICC for volume was 0.971 (95% CI, 0.957–0.981).

### 3.4. Paired Comparison and Agreement Analysis

The paired comparison between CT and pathologic maximum diameter was not statistically significant (Wilcoxon signed-rank *p* = 0.175). In the Bland–Altman analysis, the mean bias was −0.76 mm, with 95% limits of agreement from −13.66 mm to +12.13 mm. The total width of the limits of agreement was 25.79 mm.

### 3.5. Proportional Bias

**Figure 3:**
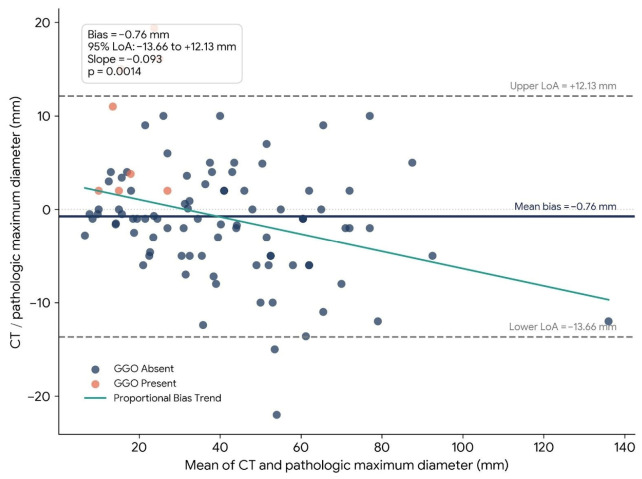
Bland–Altman plot for CT and pathologic maximum diameter. The central solid line indicates the mean bias (−0.76 mm), and the upper and lower dashed lines indicate the 95% limits of agreement (+12.13 mm and −13.66 mm, respectively). The fitted trend line demonstrates significant proportional bias, with increasing CT underestimation as tumor size increased (slope = −0.093, *p* = 0.0014).

Regression of the paired difference on the paired mean showed significant proportional bias (slope = −0.093, r = −0.321, *p* = 0.0014). Agreement and proportional bias for maximum diameter are illustrated in Figure 3.

### 3.6. GGO Subgroup Analysis

**Figure 4:**
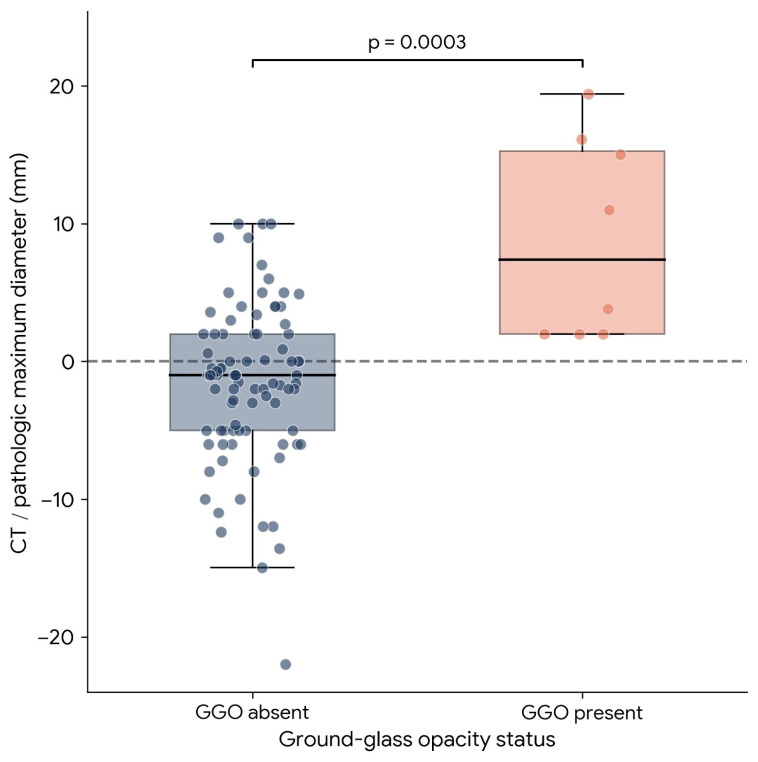
Distribution of CT/pathologic maximum diameter differences according to ground-glass opacity (GGO) status. Boxplots with overlaid individual data points show signed measurement differences (CT minus pathologic maximum diameter) in tumors without and with GGO. Tumors with GGO showed a marked shift toward CT overestimation compared with tumors without GGO (*p* = 0.0003).

In tumors without GGO, the mean CT pathology difference was −1.64 ± 5.80 mm, and the median difference was −1.00 mm. In tumors with GGO, the mean CT pathology difference was +8.91 ± 7.30 mm, and the median difference was +7.40 mm. The difference between groups was significant (Mann–Whitney U test, *p* = 0.0003). Absolute error was 4.44 ± 4.04 mm in the non-GGO group and 8.91 ± 7.30 mm in the GGO group (*p* = 0.087). In linear regression, GGO was associated with signed error (β = +10.091, *p* < 0.0001). The distribution of signed measurement differences according to GGO status is shown in Figure 4.

### 3.7. Tumor Size and Absolute Error

Across CT-based size categories, the mean absolute error was 2.57 ± 2.84 mm for tumors ≤ 20 mm, 5.60 ± 5.17 mm for tumors 20–50 mm, and 5.35 ± 4.46 mm for tumors > 50 mm. The difference in signed error across size categories was not significant (Kruskal–Wallis H = 2.57, *p* = 0.276), whereas the difference in absolute error was significant (Kruskal–Wallis H = 8.58, *p* = 0.014). In linear regression, CT maximum diameter was associated with absolute error (β = +0.065, *p* = 0.002), and GGO was also associated with absolute error (β = +5.625, *p* = 0.001).

### 3.8. Clinical Accuracy

CT maximum diameter was within ±5 mm of pathologic size in 66 of 96 cases (68.8%) and within ±10 mm in 85 of 96 cases (88.5%). In the non-GGO group, 62 of 88 cases (70.5%) were within ±5 mm and 81 of 88 cases (92.0%) were within ±10 mm. In the GGO group, 4 of 8 cases (50.0%) were within ±5 mm and 4 of 8 cases (50.0%) were within ±10 mm. Overall agreement, bias, proportional bias, and accuracy results are summarized in Table 2. Subgroup, size category, and regression results are summarized in Table 3A–C. Concordance between CT-derived and pathology-derived size-based T-categories is summarized in Table 4.

### 3.9. Size-Based T-Category Concordance Analysis

To further assess the clinical relevance of measurement discrepancies, a size-based T-category concordance analysis was performed using the size thresholds of the eighth edition of the TNM classification. CT maximum diameter was used to assign the CT-derived size-based T-category, and pathologic maximum diameter was used to assign the pathology-derived size-based T-category. The analysis was restricted to size descriptors and did not incorporate non-size T criteria. Among the 96 patients, the CT-derived and pathology-derived size-based T-categories were concordant in 60 cases (62.5%). Pathology-based upstaging relative to CT was observed in 23 cases (24.0%), and pathology-based downstaging in 13 cases (13.5%). Thus, discordance occurred in 36 of 96 patients (37.5%), with upstaging accounting for the majority of category shifts. These results are summarized in Table 4.

## 4. Discussion

In this retrospective single-center study, CT-based and pathologic tumor size measurements showed very strong correlation and excellent ICC. However, agreement at the individual patient level was more limited, as reflected by the wide Bland–Altman limits of agreement. In addition, measurement error was not constant across the size spectrum, and the presence of GGO was associated with a marked directional shift toward CT overestimation. Taken together, these findings indicate that correlation and reliability alone are insufficient to characterize the clinical performance of CT when pathologic tumor size is used as the reference standard. At the clinical level, such discrepancies may be meaningful when individual size-based decisions depend on differences measured in millimeters rather than population-level correlation alone.

This distinction is important because tumor size remains central to lung cancer staging and treatment planning. Current and proposed TNM frameworks rely heavily on size-based T classification, and imaging-based measurements are routinely used in preoperative decision-making [1,2]. In this context, a method can perform well at the cohort level while still showing clinically relevant discrepancies in individual patients. Our results support the view that CT and pathology should not be assumed to be interchangeable simply because their measurements are highly correlated.

Our findings are broadly consistent with prior studies showing discordance between radiologic and pathologic tumor size in resected lung cancer. Isaka et al. reported that CT size tended to exceed pathologic size in lung adenocarcinoma, particularly in air-containing lesions, highlighting the potential effect of non-solid components on radiologic overestimation [3]. Nagano et al. similarly demonstrated that radiologic and pathologic tumor sizes do not always match in resected NSCLC (4). More recently, Kamigaichi et al. analyzed a large multicenter cohort of early-stage NSCLC and found that radiologic whole-tumor size tended to overestimate pathologic whole-tumor size overall, while a subset of tumors showed unexpected pathological upstaging [10]. In this setting, the present study adds a more focused agreement analysis by showing that even in the absence of major mean bias, the limits of agreement can remain wide enough to matter clinically.

The Bland–Altman findings are central to the interpretation of our results. Although the mean bias was small, the 95% limits of agreement extended from approximately −13.7 mm to +12.1 mm. This degree of dispersion suggests that CT-based tumor size may provide a reasonable general estimate in many patients but remains insufficiently precise to serve as a direct surrogate for pathologic size in all cases. Prior work has also shown that apparent radiology/pathology discrepancies may reflect not only biological heterogeneity but also differences in how tumor size is measured grossly and radiologically [11,12,13]. Anderson et al. demonstrated that gross pathologic measurements can show rounding bias around whole- and half-centimeter values, which may influence pathologic T-categorization, while Hsu et al. showed that formalin fixation can alter tumor size measurements [12,13]. These considerations likely contribute to the residual disagreement observed even when correlation is strong.

A second major finding of the present study was the presence of proportional bias. CT increasingly underestimated tumor size as tumor size increased. This suggests that measurement disagreement is not uniform across the size range and may become more relevant in larger lesions. Similar concerns have been raised in radiology/pathology correlation studies of lung adenocarcinoma, where discrepancies may vary according to lesion morphology, measurement plane, and the relationship between radiologic appearance and true invasive extent [14,15]. The practical implication is that a small average bias should not be interpreted as evidence of stable performance across all tumor sizes.

Several factors may contribute to the increasing CT underestimation observed in larger tumors. From a radiologic perspective, lesion boundaries may become more difficult to define as tumor size increases, particularly when adjacent atelectasis, inflammation, or consolidation obscures the interface between tumor and surrounding lung parenchyma. Irregular morphology and differences in measurement plane may also contribute to discrepancies between radiologic and pathologic maximum diameter. From a pathologic perspective, specimen handling, fixation-related shrinkage, and gross section selection may further influence the recorded maximum diameter. Taken together, these factors suggest that the observed underestimation in larger tumors is likely multifactorial rather than attributable to a single mechanism.

The most notable finding in our cohort was the effect of GGO. In tumors with GGO, CT pathology differences shifted toward substantial CT overestimation, and clinical accuracy declined markedly. This pattern is biologically plausible and consistent with prior research on subsolid adenocarcinoma. Lee et al. showed that, in small adenocarcinomas manifesting as ground-glass nodules, the solid component on thin-section CT correlated more closely with pathologic invasion than the whole lesion diameter, whereas the overall subsolid nodule size tended to exceed pathologic tumor size [16]. Aokage et al. further supported the use of CT solid size rather than whole tumor size when correlating radiologic and pathologic invasive extent under the eighth-edition T classification [14]. Yanagawa et al. also reported that the size of the solid portion on thin-section CT correlated with pathologic invasiveness, although the exact correspondence was not perfect [17]. More recently, Ye et al., using whole-mount sections in subsolid lung adenocarcinoma, showed that radiologic/pathologic correspondence in subsolid tumors remains complex and that pathologic invasive size may be radiologically underestimated despite preserved GGO-related structure [18]. Taken together, these studies support the interpretation that GGO-containing lesions should be approached differently from purely solid tumors when CT size is compared with pathology. However, this finding should be interpreted cautiously in our cohort because the GGO subgroup was small (*n* = 8), and confirmation in larger cohorts specifically focused on GGO-containing tumors is needed.

The clinical accuracy findings in our study are aligned with this interpretation. Although CT maximum diameter fell within ±10 mm of pathologic size in most patients overall, performance was clearly less satisfactory in the GGO subgroup. This suggests that whole-lesion CT diameter may remain clinically usable as an approximate measure in many solid tumors, but caution is warranted when non-solid components are present. In such cases, the radiologic size visible on CT may reflect a composite of lepidic growth, alveolar collapse, fibrosis, and invasive tumor, rather than a straightforward morphologic equivalent of the pathologic maximum diameter [16,17,18,19].

Translating the millimetric discrepancy between CT and pathologic tumor diameter into the categorical framework of the TNM classification provides a more clinically interpretable measure of measurement error. In our cohort, the CT-derived and pathology-derived size-based T-categories were concordant in 62.5% of patients, whereas pathology yielded a higher size-based category in 24.0% and a lower category in 13.5%. These findings suggest that the observed discrepancies were not merely numerical but could also translate into stage migration in a substantial subset of patients, although this analysis was restricted to size descriptors only and did not incorporate non-size T criteria.

The secondary volumetric analysis should be interpreted more cautiously. Estimated ellipsoid CT and pathologic volumes also showed strong correlation and excellent ICC, but these data were exploratory and were derived from simple geometric estimation rather than dedicated segmentation-based volumetry. For this reason, the volumetric findings are supportive rather than definitive. They suggest that the overall radiology/pathology relationship is not limited to one-dimensional measurements, but they do not justify broader claims regarding volumetric staging or volumetric equivalence. Recent work has explored volumetric approaches to clinical T staging, but these methods differ substantially from the simplified ellipsoid estimation used in the present study and should not be considered directly comparable [20].

Although dedicated segmentation-based volumetry may provide additional morphologic information, such data were not uniformly available in this retrospective cohort. For this reason, volumetric assessment was included only as a secondary exploratory analysis based on ellipsoid estimation rather than as a primary endpoint.

Not all analytically detectable postoperative differences necessarily translate into a uniform clinical consequence, and their practical importance may depend on context rather than magnitude alone. For example, molecularly detected micrometastases may change nodal staging without consistently translating into a clear overall survival difference, although adverse prognostic implications may still emerge in selected subgroups [21].

This study has several limitations. First, it was retrospective and performed at a single center, which may limit generalizability. Second, the overall cohort was modest in size, and the GGO subgroup was particularly small (*n* = 8). Although the GGO-related effect reached statistical significance, the precision and stability of this estimate remain limited, and the finding should be confirmed in larger cohorts specifically designed to evaluate subsolid lesions. Third, pathologic size itself is not a perfectly fixed reference, because specimen handling, fixation, section selection, and gross measurement practices can influence the final recorded diameter [12,13]. Fourth, we compared maximum diameters rather than invasive size or solid component size on CT, which may be particularly relevant in subsolid lesions [14,16,17,18]. Finally, the volumetric analysis was based on estimated ellipsoid volumes rather than dedicated segmentation-based CT volumetry.

Overall, these findings support cautious interpretation of CT-derived whole-lesion diameter, particularly in GGO-containing tumors and in larger lesions, and reinforce the value of agreement-based analyses when imaging measurements are evaluated against pathology.

## 5. Conclusions

CT-based tumor size in resected lung tumors showed strong correlation and excellent reliability relative to pathologic measurements. However, agreement at the individual patient level was more limited than correlation metrics alone would suggest. GGO and tumor size appeared to be important modifiers of measurement performance, with CT tending to overestimate size in GGO-containing tumors and to increasingly underestimate size as tumor size increased; however, the GGO-related findings should be interpreted cautiously because of the small subgroup size. These findings support cautious interpretation of CT-derived whole-lesion diameter, particularly in subsolid lesions and larger tumors, and highlight the importance of agreement-based analyses when imaging measurements are evaluated against pathology.

## Figures and Tables

**Table 1:** Cohort characteristics and measurement summary.

Variable	*n*	Mean ± SD	Median (IQR)	Min	Max
CT maximum diameter, mm	96	39.24 ± 21.94	34.90 (20.85–50.73)	5.2	130.0
Pathologic maximum diameter, mm	96	40.00 ± 24.03	35.50 (20.00–55.75)	8.0	142.0
Estimated CT volume, cm^3^	96	36.01 ± 55.42	13.84 (2.57–45.58)	0.06	259.4
Estimated pathologic volume, cm^3^	96	35.89 ± 54.96	10.52 (1.88–50.43)	0.05	267.7
CT pathology difference, mm	96	−0.76 ± 6.58	−1.00 (−5.00 to 2.18)	−22.0	19.4
Absolute error, mm	96	4.82 ± 4.52	3.50 (1.68–6.00)	0.0	22.0
Percentage error, %	96	4.68 ± 32.99	−2.89 (−10.70 to 9.87)	−35.0	187.5
Categorical variables					
Variable	Value				
GGO absent, *n* (%)	88 (91.7)				
GGO present, *n* (%)	8 (8.3)				

CT maximum diameter was defined as the largest of the three orthogonal CT measurements; pathologic maximum diameter was defined similarly. Estimated volumes were calculated using the ellipsoid formula. Difference was defined as CT maximum diameter minus pathologic maximum diameter.

**Table 2:** Main agreement, bias, and accuracy results.

Outcome	Metric	Value
Primary diameter analysis	Spearman’s ρ	0.952
	ICC (95% CI)	0.959 (0.939–0.973)
	Wilcoxon signed-rank *p* value	0.175
	Mean bias, mm	−0.76
	SD of differences, mm	6.58
	Lower 95% limit of agreement, mm	−13.66
	Upper 95% limit of agreement, mm	12.13
	Width of limits of agreement, mm	25.79
	Proportional bias slope	−0.093
	Correlation between difference and mean, r	−0.321
	Proportional bias *p* value	0.0014
	Accuracy within ±5 mm, *n* (%)	66/96 (68.8)
	Accuracy within ±10 mm, *n* (%)	85/96 (88.5)
Secondary exploratory volume analysis	Spearman’s ρ	0.968
	ICC (95% CI)	0.971 (0.957–0.981)

Agreement and proportional bias metrics refer to maximum tumor diameter. Volume analysis was exploratory and based on estimated ellipsoid volumes.

**Table 3:** (**A**) GGO subgroup analysis. (**B**) CT size category analysis. (**C**) Linear regression analyses.

(**A**)				
**Variable**		**GGO Absent (*n* = 88)**	**GGO Present (*n* = 8)**	***p* Value**
CT pathology difference, mean ± SD, mm		−1.64 ± 5.80	8.91 ± 7.30	0.0003
CT pathology difference, median, mm		−1.00	7.40	0.0003
Absolute error, mean ± SD, mm		4.44 ± 4.04	8.91 ± 7.30	0.087
Accuracy within ±5 mm, *n* (%)		62/88 (70.5)	4/8 (50.0)	—
Accuracy within ±10 mm, *n* (%)		81/88 (92.0)	4/8 (50.0)	—
(**B**)				
**CT maximum diameter category**	***n***	**CT pathology difference, mean ± SD, mm**	**Median difference, mm**	**Absolute error, mean ± SD, mm**
≤20 mm	23	0.49 ± 3.56	−0.50	2.57 ± 2.84
20–50 mm	49	−0.73 ± 7.59	−1.00	5.60 ± 5.17
>50 mm	24	−2.03 ± 6.57	−1.50	5.35 ± 4.46
**Comparison**	**Statistic**	***p* value**		
Difference across size categories	Kruskal–Wallis H = 2.57	0.276		
Absolute error across size categories	Kruskal–Wallis H = 8.58	0.014		
(**C**)				
**Model**		**Predictor**	**β coefficient**	***p* value**
Signed error model		CT maximum diameter	−0.026	0.362
		GGO present	10.091	<0.0001
Absolute error model		CT maximum diameter	0.065	0.002
		GGO present	5.625	0.001

Difference was defined as CT maximum diameter minus pathologic maximum diameter. *p* values were obtained using the Mann–Whitney U test. Comparisons across CT size categories were performed using the Kruskal–Wallis test. The signed error model used CT maximum diameter minus pathologic maximum diameter as the dependent variable. The absolute error model used the absolute value of this difference. Both models included CT maximum diameter and GGO status as covariates.

**Table 4:** Concordance between CT-derived and pathology-derived size-based T-categories.

Outcome	*n* (%)
Concordant size-based T-category	60 (62.5%)
Pathology-based upstaging	23 (24.0%)
Pathology-based downstaging	13 (13.5%)
Total	96 (100.0%)

T-category comparison was based on maximum tumor diameter thresholds from the eighth edition of the TNM classification and was restricted to size descriptors only. Non-size T descriptors were not included.

## Data Availability

The data presented in this study are not publicly available because of ethical and privacy restrictions but are available from the corresponding author upon reasonable request.

## Abbreviations

The following abbreviations are used in this manuscript:

CT	Computed tomography
GGO	Ground-glass opacity
ICC	Intraclass correlation coefficient
LoA	Limits of agreement
SD	Standard deviation
IQR	Interquartile range
CI	Confidence interval
TNM	Tumor, node, metastasis

## References

[1] Rami-Porta R., Bolejack V., Crowley J., Ball D., Kim J., Lyons G., Rice T., Suzuki K., Thomas C.F., Travis W.D. (2015). The IASLC lung cancer staging project: Proposals for the revisions of the T descriptors in the forthcoming eighth edition of the TNM classification for lung cancer. J. Thorac. Oncol..

[2] Detterbeck F.C., Woodard G.A., Bader A.S., Dacic S., Grant M.J., Park H.S., Tanoue L.T. (2024). The proposed ninth edition TNM classification of lung cancer. Chest.

[3] Isaka T., Yokose T., Ito H., Imamura N., Watanabe M., Imai K., Nishii T., Woo T., Yamada K., Nakayama H. (2014). Comparison between CT tumor size and pathological tumor size in frozen section examinations of lung adenocarcinoma. Lung Cancer.

[4] Nagano T., Takamori S., Hashinokuchi A., Matsydo K., Kohno M., Miura N., Takenaka T., Kamitani T., Shimokawa M., Ishigami K. (2023). Comparison of radiological and pathological tumor sizes in resected non-small cell lung cancer. Gen. Thorac. Cardiovasc. Surg..

[5] Bland J.M., Altman D.G. (1986). Statistical methods for assessing agreement between two methods of clinical measurement. Lancet.

[6] Giavarina D. (2015). Understanding Bland Altman analysis. Biochem. Medica.

[7] Erasmus L.T., Strange T.A., Agrawal R., Strange C.D., Ahuja J., Shroff G.S., Truong M.T. (2023). Lung cancer staging: Imaging and potential pitfalls. Diagnostics.

[8] McGraw K.O., Wong S.P. (1996). Forming inferences about some intraclass correlation coefficients. Psychol. Methods.

[9] Koo T.K., Li M.Y. (2016). A guideline of selecting and reporting intraclass correlation coefficients for reliability research. J. Chiropr. Med..

[10] Kamigaichi A., Tsutani Y., Mimae T., Miyata Y., Adachi H., Shimada Y., Takeshima Y., Ito H., Ikeda N., Okada M. (2024). Discrepancy between radiological and pathological tumor size in early-stage non-small cell lung cancer: A multicenter study. Semin. Thorac. Cardiovasc. Surg..

[11] Lampen-Sachar K., Zhao B., Zheng J., Moskowitz C.S., Schwartz L.H., Zakowski M.F., Rizvi N.A., Kris M.G., Ginsberg M.S. (2012). Correlation between tumor measurement on computed tomography and resected specimen size in lung adenocarcinomas. Lung Cancer.

[12] Anderson K.R., Heidinger B.H., Chen Y., Bankier A.A., VanderLaan P.A. (2017). Measurement bias of gross pathologic compared with radiologic tumor size of resected lung adenocarcinomas. Am. J. Clin. Pathol..

[13] Hsu P.K., Huang H.C., Hsieh C.C., Hsu H.S., Wu Y.C., Huang M.H., Hsu W.-H. (2007). Effect of formalin fixation on tumor size determination in stage I non-small cell lung cancer. Ann. Thorac. Surg..

[14] Aokage K., Miyoshi T., Ishii G., Kusumoto M., Nomura S., Katsumata S., Sekihara K., Hishida T., Tsuboi M. (2017). Clinical and pathological staging validation in the eighth edition of the TNM classification for lung cancer: Correlation between solid size on thin-section computed tomography and invasive size in pathological findings in the new T classification. J. Thorac. Oncol..

[15] Heidinger B.H., Anderson K.R., Moriarty E.M., Costa D.B., Gangadharan S.P., VanderLaan P.A., Bankier A.A. (2017). Size measurement and T-staging of lung adenocarcinomas manifesting as solid nodules ≤30 mm on CT. Acad. Radiol..

[16] Lee K.H., Goo J.M., Park S.J., Wi J.Y., Chung D.H., Go H., Park H.S., Park C.M., Lee S.M. (2014). Correlation between the size of the solid component on thin-section CT and the invasive component on pathology in small lung adenocarcinomas manifesting as ground-glass nodules. J. Thorac. Oncol..

[17] Yanagawa M., Kusumoto M., Johkoh T., Noguchi M., Minami Y., Sakai F., Asamura H., Tomiyama N., Awai K., Minami M. (2018). Radiologic-pathologic correlation of solid portions on thin-section CT images in lung adenocarcinoma: A multicenter study. Clin. Lung Cancer.

[18] Ye T., Shen X., Wang S., Wu H., Wang Y., Hu H., Zhang Y., Huang Q., Wang Z., Gu Y. (2025). Study of the radiologic and pathologic correlations for subsolid lung adenocarcinoma with the application of whole-mount sections (ECTOP1011). Transl. Lung Cancer Res..

[19] Heidinger B.H., Anderson K.R., Nemec U., Costa D.B., Gangadharan S.P., VanderLaan P.A., Bankier A.A. (2017). Lung adenocarcinoma manifesting as pure ground-glass nodules: Correlating CT size, volume, density, and roundness with histopathologic invasion and size. J. Thorac. Oncol..

[20] Sayan M., Kankoc A., Aslan M.T., Akarsu I., Kurul İ.C., Celik A. (2025). Recommendation for clinical T staging in patients with non-small cell lung cancer: Volumetric measurement: A retrospective study from Turkey. J. Chest Surg..

[21] Süer H., Erus S., Cesur E.E., Yavuz Ö., Ağcaoğlu O., Bulutay P., Önder T.T., Tanju S., Dilege Ş. (2023). Combination of CEACAM5, EpCAM and CK19 gene expressions in mediastinal lymph node micrometastasis is a prognostic factor for non-small cell lung cancer. J. Cardiothorac. Surg..

